# Oscillatory Shear Stress-Induced Arginase Activity May Explain Reduced Exhaled Nitric Oxide Levels after Vest Chest Physiotherapy in Cystic Fibrosis

**DOI:** 10.1155/2014/725317

**Published:** 2014-04-03

**Authors:** Christoph Nowak

**Affiliations:** Division of Pulmonology, University Hospital Zurich, Raemistra*β*e 100, 8091 Zurich, Switzerland

The well-executed study by Sisson et al. [1] demonstrates the potential of vest chest physiotherapy (VCPT) in improving airway clearance in cystic fibrosis (CF) patients. The authors point out that they observed a nonsignificant tendency towards higher exhaled nitric oxide (NO_*x*_) levels in CF compared to healthy control subjects which is at odds with previous findings of lower NO_*x*_ in CF [2, 3]. Contrary to Sisson et al.'s hypothesis, VCPT in CF patients reduced rather than increased NO_*x*_. The authors discuss this unexpected finding with regard to increased cellular NO utilisation and distal airway mucous barriers to NO diffusion.

An alternative explanation involves NO synthetase (NOS) and arginase activity in airway epithelial cells. Both enzymes compete for L-arginine as substrate and imbalances in activity levels are believed to be important in the pathogenesis of airway inflammation and hyperreactivity [4]. Arginase converts L-arginine into L-ornithine and urea, effectively modulating NOS activity, proinflammatory oxidant species generation, and airway remodelling [5]. Impaired lung function in CF is likely associated with reduced NO-mediated bronchodilation arising from low levels of L-arginine, impaired NOS expression, and increased arginase activity [6, 7]. Elevated systemic arginase and concomitantly reduced L-arginine levels were observed in acutely hospitalised CF patients and normalised after successful treatment [8]. Increased arginase activity, in addition to limiting NO production, also contributes to CF lung pathology through downstream products [9], is associated with Pseudomonas infection, and inversely related to lung function [10]. Arginase is moreover thought to play a role in the pathogenesis of other lung diseases like asthma and chronic obstructive pulmonary diseases [11, 12]. Recently, inhaled L-arginine was demonstrated to be a safe and potentially beneficial treatment approach in CF [13].

A possible mechanism to explain Sisson et al.'s [1] surprising finding of lower NO_*x*_ after VCPT may be an increase in arginase activity through oscillatory mechanical stimulation of lung epithelial cells. Several studies in animal and cell models have demonstrated that specific patterns of mechanical stimulation can induce vascular endothelial arginase expression and activity. For instance, oscillatory shear stress induced stronger arginase activation than application of unidirectional shear force in porcine [14] and ApoE-deficient murine carotid artery segments [15]. An* in vitro* study showed that cyclic stretching of vascular smooth muscle cells upregulated arginase mRNA expression and enzyme activity whilst inhibiting NOS [16]. The VCPT device used in Sisson et al.'s [1] study produced oscillatory inflation/deflation cycles at a rate of 10–15 Hz throughout 20-minute treatment sessions. Thus, a possible explanation for reduced exhaled NO could be oscillatory shear stress-induced upregulation of arginase activity and associated inhibition of NOS. Whilst the observed vascular endothelial response in arginase activity to shear forces remains to be demonstrated for airway epithelial cells, the suggested link provides a plausible theory to account for Sisson et al.'s intriguing findings.
